# The role of S100 proteins in the pathogenesis and monitoring of autoinflammatory diseases

**DOI:** 10.1186/s40348-018-0085-2

**Published:** 2018-09-25

**Authors:** Dirk Holzinger, Dirk Foell, Christoph Kessel

**Affiliations:** 1 Department of Pediatric Hematology-Oncology, University of Duisburg-Essen, Hufelandstr. 55, 45122 Essen, Germany; 2 Department of Pediatric Rheumatology and Immunology, University Children’s Hospital Muenster, Domagkstr. 3, 48149 Muenster, Germany

**Keywords:** S100 proteins, Autoinflammation, DAMP, Biomarker, Fever of unknown origin, Diagnosis, Monitoring, TLR agonist, Calgranulins

## Abstract

S100A8/A9 and S100A12 are released from activated monocytes and granulocytes and act as proinflammatory endogenous toll-like receptor (TLR)4-ligands. S100 serum concentrations correlate with disease activity, both during local and systemic inflammatory processes. In some autoinflammatory diseases such as familial Mediterranean fever (FMF) or systemic juvenile idiopathic arthritis (SJIA), dysregulation of S100 release may be involved in the pathogenesis. Moreover, S100 serum levels are a valuable supportive tool in the diagnosis of SJIA in fever of unknown origin. Furthermore, S100 levels can be used to monitor disease activity to subclinical level, as their serum concentrations decrease with successful treatment.

## Functions of phagocyte-specific S100 proteins

The S100 protein family represents the largest subgroup within the Ca^2+^-binding EF-hand protein superfamily. Constitutive expression of the phagocyte-specific S100 proteins A8 (also termed calgranulin or myeloid-related protein, MRP8) and A9 (calgranulin B, MRP14) as well as A12 (calgranulin C, MRP6) is largely restricted to granulocytes and monocytes while S100A12 is only expressed by human neutrophils [33].

While a number of different intracellular mechanistic implications have been proposed for S100A8/A9 (reviewed in [2]), very little data suggest an intracellular function of S100A12 (Table 1).

**Table 1:** Intracellular calgranulin functions

	S100A8/A9	S100A12
Neutrophils	- Ca^2+^ store/sensor [2] - ↑Phagocytosis [20] - ↑ROS [31], S100A8: ↓ROS [23] - Ca^2+^-dependent interaction with cytoskeleton [27, 29, 32]: ↑migration, ↑degranulation, ↑phagocytosis - S100A9 ↓microtubule polymerization [32]	- Zn^2+^-homeostasis? [26]
Monocytes	- Ca^2+^ store/sensor [2] - Ca^2+^-dependent interaction with cytoskeleton [27, 29, 32]: ↑migration, ↑degranulation, ↑phagocytosis - S100A9 ↓microtubule polymerization [32]	

**Fig. 1:**
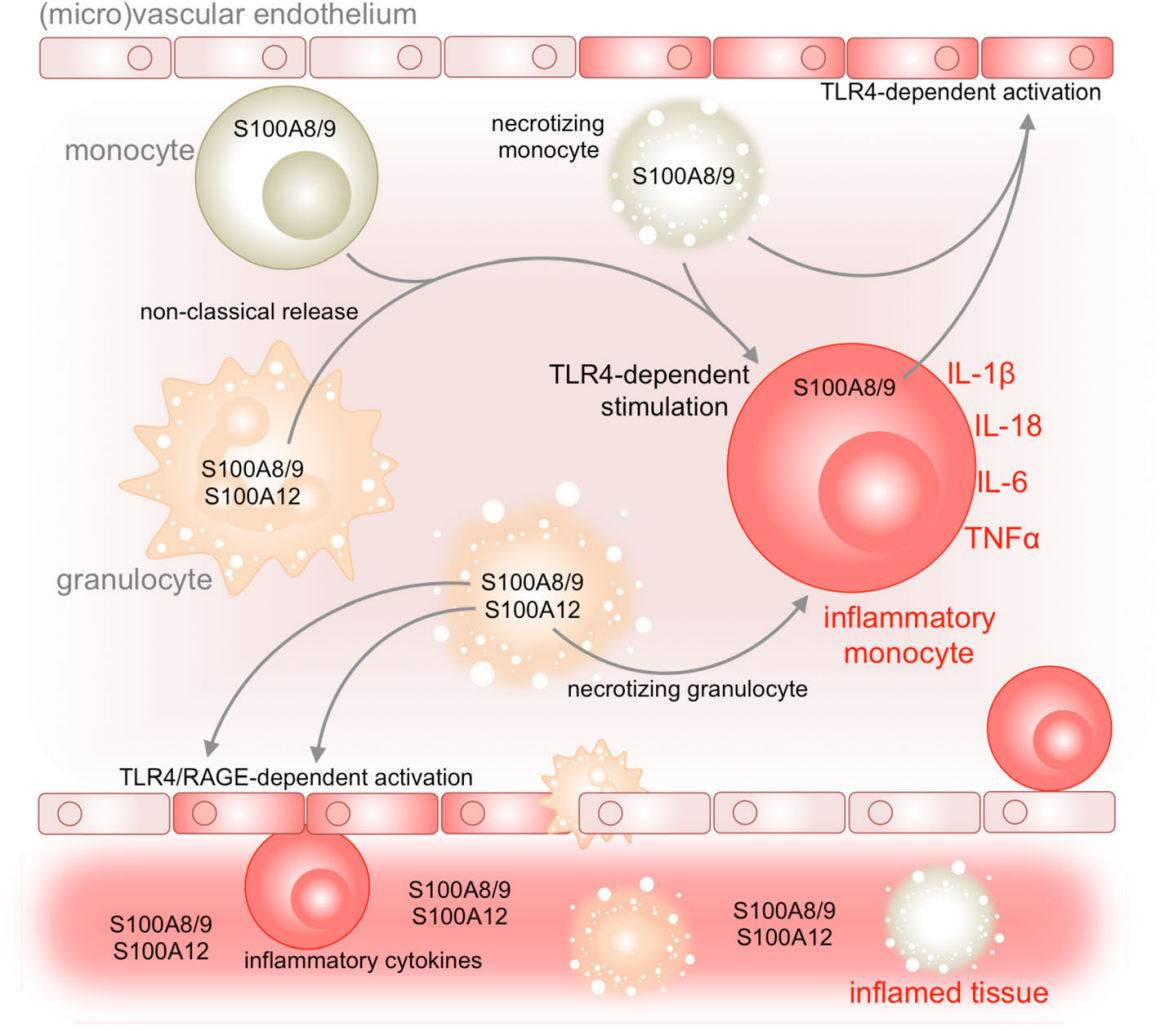
DAMP functions of calgranulins. Calgranulins can be released by circulating neutrophils (S100A8/A9 and S100A12) or monocytes (S100A8/A9) upon cellular necrosis or active, non-classical transport. Once, extracellular calgranulins can trigger proinflammatory activation of human monocytes in a toll-like receptor 4 (TLR4)-dependent manner. Via sensors such as the multi-ligand receptor for advanced glycation end products or TLR4, S100A8/A9 and A12 can further induce proinflammatory activation of vascular endothelium, which facilitates leukocyte rolling and subsequent extravasation, and thus promotes tissue inflammation

S100A8, A9, and A12 are lacking structural elements required for secretion via the classical endoplasmic reticulum and Golgi-dependent secretory pathway. Thus, one of the primary, though passive, release “mechanisms” involves necrotic cell death. Further, there is evidence for active cytoskeleton-dependent non-classical secretion [5, 27, 32] (Fig. 1), which is similarly used by cytokines such as interleukin (IL)-1 [30].

Once released from cells, the extracellular role of calgranulins as damage-associated molecular patter (DAMP) molecules is potentially most relevant in the context of autoinflammation (Fig. 1). In this respect, a majority of studies limits receptor binding and inflammatory signaling of calgranulins to toll-like receptor 4 (TLR4) [5, 16, 17, 24, 28].

## Role of S100 proteins in autoinflammatory diseases

Hypersecretion of S100 proteins can result in a sterile inflammatory environment, which triggers proinflammatory cytokine as well as further S100 expression [9, 15] (Fig. 1). During inflammatory attacks, serum levels of S100 proteins are massively elevated in FMF and the excessive amount of these proteins suggests its involvement in the pathogenesis this disease [9, 11]. Pyrin, which is mutated in FMF, interacts with PSTPIP1, which causes pyogenic sterile arthritis, pyoderma gangrenosum, and acne (PAPA) syndrome and PSTPIP1-associated myeloid-related proteinemia inflammatory (PAMI) [13]. Especially the latter shows excessively high S100 levels [11]. S100A8 and A9 bind to both the subcellular actin network and microtubules [32], which might link these proteins to pyrin and PSTPIP1. Accordingly, colchicine, which is effective in FMF and blocks tubulin-dependent processes, inhibits alternative secretion of S100 proteins [25].

The predominant role of the innate immune system in SJIA is underscored by high serum concentrations of S100 proteins. These concentrations are closely associated with disease activity and can be found neither in other forms of inflammatory arthritis nor in other autoimmune or infectious diseases [3, 4, 8]. Furthermore, extracellular S100A8 and S100A9 form a positive inflammatory feedback loop with IL-1ß, and depletion of these proteins from SJIA patient’s serum diminishes the IL-1ß-inducing capacity of this serum [7].

In contrast, in the cryopyrin-associated periodic syndromes (CAPS) or periodic fever, aphthous stomatitis, pharyngitis, adenitis syndrome (PFAPA) S100 levels are within the range of those found in infectious diseases. Although the exact role of the S100 proteins in CAPS has not yet been fully understood, these proteins are promising markers of IL-1ß-driven inflammation [21]. In PFAPA, S100 proteins are upregulated during flares and are within the range of healthy controls during symptom-free intervals [18].

## S100 proteins as biomarkers in clinical practice

Fever of unknown origin (FUO) is a challenging medical problem predominantly caused by infections, malignancies, immunodeficiency syndromes, and autoimmune or autoinflammatory diseases [1]. S100A8/A9 and S100A12 levels can potentially differentiate SJIA from other causes of FUO including systemic infections but not FMF [6, 7, 34]. The third disease group that shows constantly extremely elevated S100 protein serum levels is PAPA/PAMI [11] (Table 2).

**Table 2:** Serum concentration of phagocyte-specific S100 proteins in systemic inflammatory diseases (adapted and updated from [15])

	S100A8/A9 levels (ng/ml)	*N*	S100A12 levels (ng/ml)	*N*
Healthy controls	340 ± 70	50	50 ± 10	45
			50 (5)**	74
Monogenic autoinflammatory diseases				
*FMF*	*110,000 ± 82,000*	*20*	*6720 ± 4960*	*17*
			33,500 (22,200)**	*7*
*PAPA*	*116,000 ± 74,000*	*11*	*–*	
*PAMI*	*2,045,000 ± 1,300,000*	*13*	*–*	
NOMID	2830 ± 580	18	720 ± 450	18
MWS	4390 (2535)*	12	150 ± 60	17
FCAS	3600 (4610)*	5	–	–
Polygenic autoinflammatory diseases				
*Systemic-onset JIA*	*14,920 ± 4030*	*60*	*7190 ± 2690*	*60*
	*24,750 ± 11,410*	*20*	3700 (1080)**	*33*
Polyarticular JIA	2380 ± 530	89	395 (45)**	89
PFAPA	3846 ± 1197	15	685 ± 210	15
Vasculitis				
Kawasaki disease	3630 ± 480	21	398 (294)*	67
Henoch-Schoenlein nephritis	881 ± (670)*	30	–	–
Infections				
Severe febrile infections	3720 ± 870	66	470 ± 160	83

All other data are mean ± 95% confidence interval

Italics indicate the diseases with the significantly highest S100 protein serum levels

*FCAS* familial cold autoinflammatory syndrome, *FMF* familial Mediterranean fever, *JIA* juvenile idiopathic arthritis, *MWS* Muckle-Wells syndrome, *N* number of patients, *NOMID* Neonatal Onset Multisystem Inflammatory Disorder, *PAMI* PSTPIP1-associated myeloid-related proteinemia inflammatory, *PAPA* pyogenic sterile arthritis, pyoderma gangrenosum, and acne syndrome, *PFAPA* periodic fever, aphthous stomatitis, pharyngitis, adenitis syndrome

*Mean (standard deviation)

**Mean (standard error of the mean)

In patients with an established diagnosis of an autoinflammatory disorder, rapid commencement of effective therapy is essential to avoid damage and complications. In autoinflammatory diseases, acute phase reactants are commonly elevated, including SAA and CRP as markers of inflammation [10]. As a more sensitive biomarker, S100A12 has been demonstrated to reflect clinical disease activity and therapeutic response in MWS [19]. Various states of subclinical disease activity were demonstrated in all types of CAPS, depending on the type of anti-IL-1 therapy. Here, S100A8/A9 proved to be a sensitive biomarker for monitoring disease activity and response to IL-1 blockade [35]. In FMF, S100A12 shows an excellent correlation to disease activity [14, 34]. S100A12 may also allow stratification of FMF patients according to disease severity [9]. Moreover, S100A12 reflects subclinical inflammation in heterozygous carriers of MEFV gene mutations, and patients with well controlled anti-inflammatory treatment have significantly decreased serum levels [22]. The same applies for SJIA, where S100A8/A9 serum concentrations correlate closely with response to treatment and disease activity [12]. In SJIA, S100A8/A9 serum concentrations are the first predictive biomarker indicating subclinical disease activity and stratifying patients at risk of relapse during times of clinically inactive disease [12].

S100A8/A9 and S100A12 can thus be used as surrogate markers not only to monitor therapeutic responses at initiating therapies with the goal of inducing remission, but also during maintenance therapies.
